# Regulation of Intestinal Butyrate Transporters by Oxidative and Inflammatory Status

**DOI:** 10.3390/antiox15010048

**Published:** 2025-12-30

**Authors:** Fátima Martel

**Affiliations:** 1 Unit of Biochemistry, Department of Biomedicine, Faculty of Medicine, University of Porto, 4200-319 Porto, Portugal; fmartel@med.up.pt; 2 Instituto de Investigação e Inovação em Saúde (i3S), University of Porto, 4200-135 Porto, Portugal

**Keywords:** oxidative stress, inflammation, butyrate transporters, intestine, colorectal cancer, inflammatory bowel disease

## Abstract

Beneficial effects of the microbiota-derived metabolite butyrate at the colonic level are well established, particularly through its relevance in colorectal cancer (CRC) and inflammatory bowel disease (IBD), two major intestinal pathologies. Therefore, the mechanisms involved in butyrate transport across colonic epithelial cell membranes (uptake transporters: monocarboxylate transporter 1 (MCT1) and sodium-coupled monocarboxylate transporter 1 (SMCT1); efflux transporters: breast cancer resistance protein (BCRP) and MCT1/monocarboxylate transporter 4 (MCT4)), which are determinant for its intracellular levels, are of primary importance for its beneficial effects at the colonic level. The available data suggest that all these butyrate transporters can be modulated by redox and inflammatory status, but the evidence is scarce and rather inconsistent. Nevertheless, a role of nuclear factor erythroid 2-related factor 2 (Nrf2) and of the proinflammatory cytokines tumor necrosis factor-α (TNF-α) and interferon-γ (IFN-γ) in mediating the effect of oxidative stress and inflammation, respectively, on MCT1 and SMCT1 is suggested. So, more investigation on this subject is needed, given the fact that increased oxidative stress levels and inflammatory status are present in a series of intestinal conditions and pathologies, including CRC and IBD, which could help to establish these transporters as potential cellular targets in these diseases.

## 1. Introduction—The Role of Butyrate in the Human Intestine

Butyrate, along with the two other short-chain fatty acids (acetate and propionate), is a product of intestinal fermentation of dietary fiber by the microbiota and plays a crucial role in maintaining colonic epithelial homeostasis. Butyrate exerts multiple regulatory and beneficial effects within the colon, including the fact that this short-chain fatty acid: (1) is the preferred energy substrate for colonocytes; (2) stimulates growth and proliferation of normal intestinal epithelial cells; (3) inhibits inflammation; (4) inhibits oxidative stress; (5) improves the defense barrier function; (6) stimulates mucus secretion; (7) stimulates fluid and electrolyte absorption; and (8) inhibits colon carcinogenesis [1,2].

The beneficial actions of butyrate at the intestinal level result from its effect on epithelial and immune cells resulting from two distinct, but complementary, pathways: binding to G-protein-coupled receptors (GPR41, GPR43, and GPR109A) and inhibition of histone deacetylases (HDACs), thereby influencing gene transcription [1,2]. Therefore, the mechanisms involved in BT transport across biological membranes at the intestinal level (including both uptake and efflux mechanisms) and their regulation are very relevant in the context of the beneficial effects of this compound at the colonic level.

## 2. Membrane Transport of Butyrate in the Intestinal Epithelium

Undissociated butyrate can passively diffuse across the lipid membrane of colonocytes. However, since more than 90% of butyrate (pKa of ≅ 4.8) is ionized at luminal pH (≅6.0), this mechanism plays a very limited physiological role in its transport. Instead, transport of this compound across intestinal cell membranes is mainly mediated by transport systems [2,3,4,5,6]. At the intestinal level, two major monocarboxylate transporters, belonging to distinct transporter families, have been identified for butyrate absorption across the luminal membranes of colonocytes. These transporters are the proton-coupled electroneutral monocarboxylate transporter 1 (MCT1, SLC16A1) (H^+^: butyrate stoichiometry; 1:1) and the sodium-coupled electrogenic monocarboxylate transporter 1 (SMCT1, SLC5A8) (Na^+^: butyrate stoichiometry; 2:1) [2,3,4,5,6].

Additionally, MCT1 and MCT4 (SLC16A3; proton-coupled electroneutral monocarboxylate transporter 4) (H^+^: butyrate stoichiometry; 1:1), another transporter belonging to the SLC16 family, were identified in the basolateral membrane of colonic epithelium and may be involved in butyrate efflux from the colonocytes into the serosal side [6].

Moreover, butyrate is also a substrate of ABCG2 (also known as BCRP or breast cancer resistance protein), an ATP-dependent efflux pump expressed in the lumen-facing apical membrane of colonic epithelial cells, able to transport butyrate from the colonocytes into the intestinal lumen [7].

To summarize, butyrate is absorbed from the colon lumen into colonocytes by both MCT1- and SMCT1-mediated transport and can be apically and basolaterally removed from these cells, by BCRP- and MCT1/4-mediated mechanisms, respectively.

## 3. Regulation of Butyrate Transport by Oxidative Stress/Redox Levels

Increased reactive oxygen species (ROS) levels, also known as oxidative stress, are a result of either increased ROS generation and/or a loss of antioxidant defense mechanisms. A major consequence of oxidative stress is damage of tissue via direct oxidation of nucleic acid bases, lipids, and proteins, but also via profound alterations in signal transduction pathways, which can severely compromise cellular functions. Not surprisingly, it is associated with numerous pathologies, from atherosclerosis to neurodegenerative diseases, inflammation, and cancer [8,9,10,11].

**Figure 1:**
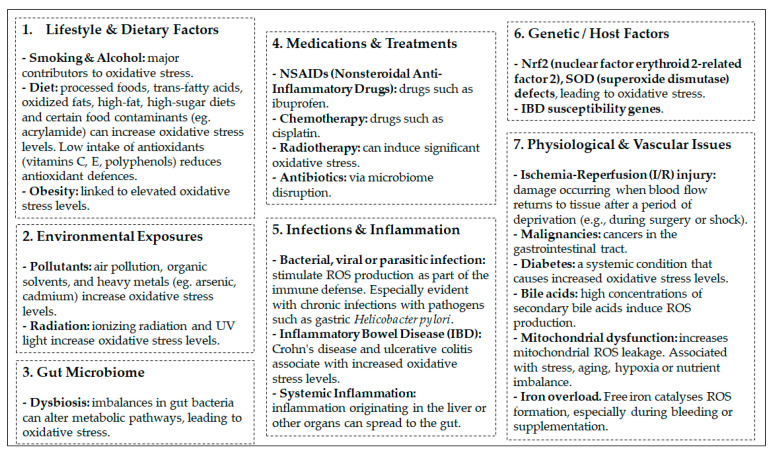
Major causes of intestinal oxidative stress.

The gastrointestinal tract is a major target for oxidative stress damage due to constant exposure to ROS generated not only by endogenous (cellular) sources but also by a large variety of xenobiotics (air pollutants, tobacco smoke, ionizing and non-ionizing radiations, heavy metals, foods, and drugs), endogenous toxic substances (e.g., bile acids), as well as by microbial and microbiota metabolism [12,13]. An imbalance in the cellular redox system leading to increased levels of ROS is associated with many gastrointestinal tract conditions or diseases, including ischemia/reperfusion, inflammatory bowel disease, surgical stress, radiation enteritis, iron supplementation, Zn deficiency, methotrexate therapy, and colorectal cancer (CRC) [12,14] (Figure 1).

Oxidative stress, and ROS in particular, can modulate transporter function by a number of mechanisms, including: (a) regulation of the expression of transporters at transcriptional, posttranscriptional, and epigenetic levels; (b) regulation of the biosynthesis of cofactors required for the transporter’s function; (c) posttranslational modifications of transporter structure/conformational changes in the transporter (e.g., formation of intra- or interprotein crosslinked derivatives, peptide cleavage, nitrosylation, nitration, and oxidation of key amino acid residues with production of hydroxyl or carbonyl derivatives); and (d) amplification of the copy number of genes encoding these transporters [15,16].

Although oxidative stress is a factor affecting the function and expression of many intestinal transporters [15,17], not much is known concerning the effect of an oxidative environment on intestinal butyrate transport (Table 1).

Our group investigated the effect of *tert*-butylhydroperoxide (*t*BOOH)-induced oxidative stress on butyrate uptake by rat intestinal epithelial cells (IEC-6 cells). We verified that *t*BOOH strongly reduced total and Na^+^-dependent butyrate uptake, but because it did not affect Na^+^-independent uptake, we concluded that it strongly reduced SMCT1-mediated, but not MCT1-mediated, butyrate uptake by these cells. The lack of effect of oxidative stress on MCT1 was substantiated by the fact that *t*BOOH did not interfere with butyrate uptake by Caco-2 cells, a human colorectal adenocarcinoma cell line in which butyrate uptake is mainly MCT1-mediated. Moreover, *t*BOOH increased uptake and efflux of butyrate by passive diffusion. We could also conclude, by using specific NADPH and xanthine oxidases inhibitors, that the inhibitory effect of oxidative stress upon SMCT1-mediated butyrate transport was dependent on the generation of ROS by these two enzymes. Moreover, we also verified that the inhibitory effect of oxidative stress upon SMCT1-mediated butyrate transport was not related to changes in either *SMCT1* transcription rates or its membrane insertion. Rather, by using specific kinase inhibitors, we concluded that this inhibitory effect was dependent on posttranslational regulation by ERK1/2 and tyrosine kinase intracellular pathways. In contrast, it was not affected by specific inhibitors of protein kinase A, protein kinase C, Ca^2+^/calmodulin-dependent protein kinase II, c-Jun N-terminal kinase (JNK), and p38 MAPK. Importantly, the negative effect of oxidative stress upon butyrate uptake was partially prevented by quercetin and resveratrol, two dietary polyphenols with antioxidant properties [18].

In another work, we verified that the bile salt chenodeoxycholic acid was able to reduce both MCT1- and SMCT1-mediated butyrate uptake by IEC-6 cells. Interestingly, although the ROS scavenger *N*-acetylcysteine did not alter butyrate uptake, it was able to partially reduce the inhibitory effect of chenodeoxycholic acid, suggesting that the inhibition of butyrate transport by this bile salt is at least partly related to its pro-oxidant effect [19].

However, a contrasting effect was observed with another oxidative stress inducer, *tert*-butylhydroquinone (*t*BHQ). In both normal (non-cancerous) and cancerous intestinal epithelial cell lines (NCM460 and HCT15 cells, respectively), this agent led to an increase in *MCT1* mRNA and protein levels (together with a decrease in MCT4 levels). Interestingly, the effect of *t*BHQ on MCT1 was associated with increased expression of Nrf2, a transcription factor that has a key role in the cellular response to oxidative stress, by inducing the expression of numerous antioxidant and detoxifying enzymes as well as genes involved in cell growth and survival. Treatment of NCM460 cells with small interfering RNA against Nrf2 decreased *MCT1* expression, confirming the role of Nrf2 in *MCT1* regulation in the colon [20] (Table 1).

The stimulating effect of Nrf2 activation on MCT1 appears not to be restricted to the intestine. Indeed, a similar Nrf2-dependent MCT1 activation was also described in C57BL/6 J mice, where administration of the Nrf2 activator sulforaphane led to an increase in *MCT1* mRNA and protein levels in skeletal muscle. In contrast, the expression of *MCT4* mRNA decreased after pretreatment with this compound, although it did not affect MCT4 protein expression [21]. Similarly, oxidative stress caused by glutamine depletion led to an Nrf2-dependent induction of *MCT1* expression in pancreatic ductal adenocarcinoma T3M4 and A818-6 cell lines [22].

A contrasting effect of different oxidative stress generators on butyrate transporters was also verified in an amyotrophic lateral sclerosis (ALS) cell line model (a mutant motor neuron-like NSC-34 cell line). The authors verified that exposure of these cells to H_2_O_2_ or glutamate, two conditions that increase oxidative stress levels, led to opposite effects (increase and decrease, respectively) on *SMCT1* mRNA levels and activity [23].

Finally, in the SiHa human cervix squamous carcinoma cell line, the *MCT1* gene and protein expression were increased by the ROS generator H_2_O_2_, and this overexpression was blocked by both the antioxidant *N*-acetyl-L-cysteine and the mitochondria-selective superoxide scavenger MitoTEMPO [24].

Very little is known concerning the effect of redox state on BCRP-mediated butyrate transport at the intestinal level. The only study on this subject concluded that *t*BOOH does not interfere with BCRP-mediated butyrate efflux from IEC-6 cells [18]. Nevertheless, the consequences of oxidative stress on intestinal BCRP levels have been investigated in a few studies, although the results are not straightforward. While some oxidative stress inducers (doxorubicin alone or in combination with CoCl) were reported to increase BCRP protein levels in wild-type and doxorubicin-resistant HT-29 cells [25], a reduction in BCRP levels was found with other oxidative stress inducers (e.g., inosine, hypoxanthine, and hypericin + 6 h photodynamic therapy) in rat ileum and human colon adenocarcinoma Caco-2 and HT-29 cell lines [26,27]. The same discrepant effect of distinct oxidative stress inducers on BCRP in other organs has also been found [28,29,30,31].

Overall, we conclude that different oxidative stress inducers appear to induce distinct changes in MCT1-, SMCT1-, and BCRP-mediated butyrate transport both at the colonic and extra-colonic level. The discrepancy may be related to differences in the mechanisms involved in generating increased oxidative stress levels (such as differences in the ROS species generated or in the ROS-generating mechanism) or differences in the modulation of antioxidant defenses. Moreover, it can also result from the fact that the effect of oxidative stress inducers was frequently evaluated by using compounds with many other biological actions.

**Table 1:** Effect of increased oxidative stress levels and inflammation on butyrate transporters at the intestinal level. Symbols: ↑ increase, ↓ decrease, = no effect, - not determined.

Transporter	Oxidative Stress Inducer(s)	Experimental Model	Observed Effect	Mechanism	Ref.
SMCT1	tBOOH (3 mM; 1 h)	IEC-6 cells	↓	Decrease in SMCT1-mediated butyrate uptake; no effect on MCT1-mediated uptake No effect on SMCT1 mRNA level No effect on SMCT1 membrane insertion rate Posttranslational (dependent on regulation by ERK1/2 and tyrosine kinase intracellular pathways)	[18]
MCT1		Caco-2 cells	=	No effect on MCT1-mediated uptake	
MCT1	Chenodeoxycholic acid (100 µM; 48 h)	IEC-6 cells	↓	Decrease in MCT1- and SMCT1-mediated butyrate uptake No effect on MCT1 mRNA levels	[19]
SMCT1			↓	Increase in SMCT1 mRNA levels	
MCT1	tBHQ (50 µM; 24–72 h)	NCM460 cells, HCT15 cells	↑	Increase in MCT1 mRNA and protein levels This effect is Nrf2 activation-dependent	[20]
MCT4			↓	Decrease in MCT4 mRNA and protein levels	
BCRP	tBOOH (3 mM; 1 h)	IEC-6 cells	=	-	[18]
BCRP	Doxorubicin (5 µM; 24 h) CoCl (100 µM; 24 h)) (chemically induced hypoxia) Doxorubicin (5 µM) + CoCl (100 µM) (24 h)	HT-29wt cells, HT-29DxR (doxorubicin-resistant) cells	↑	Increase in BCRP protein levels ROS-dependent	[25]
BCRP	Inosine (2% in diet; 24 h)	Rat ileum	↓	No change in BCRP mRNA and protein levels Decrease in BCRP homodimer levels Dependent on xanthine-oxidase-derived ROS	[26]
	Hypoxanthine (1–5 mM; 3 h)	Caco-2 cells	↓	No change in BCRP mRNA and protein levels Decrease in BCRP homodimer levels Dependent on xanthine-oxidase-derived ROS	
BCRP	Hypericin (50 nM) (16 h) + 6h Photodynamic therapy Hypericin (50 nM) + Hyperforin (1–5 µM) (16 h) + 6h Photodynamic therapy Hypericin (50 nM) + Aristoforin (1–5 µM) (16 h) + 6h Photodynamic therapy	HT-29 cells	↓	Decrease in BCRP mRNA and protein levels	[27]
**Transporter**	**Experimental Model/** **Inflammatory Mediator**		**Observed Effect**	**Mechanism**	**Ref.**
MCT1	Inflamed colonic mucosa of patients with active UC (vs. control subjects)		↓	Decrease in butyrate uptake and oxidation Decrease in MCT1 mRNA levels	[32]
MCT1	Inflamed colonic mucosa of patients with active UC (vs. non-inflamed mucosa and control subjects)		↓	Decrease in MCT1 and BCRP mRNA levels The decrease in MCT1 and BCRP levels is > than in non-inflamed colonic mucosa	[33]
BCRP			↓/=	Decrease in protein levels of MCT1 No change in protein levels of BCRP	
MCT1	Inflamed colonic mucosa of patients with active UC and CD (vs. control subjects)		↓	Decrease in MCT1 and BCRP mRNA and protein levels	[34]
BCRP	Ex vivo differentiated epithelial organoids from healthy individuals/TNF-α (50 ng/mL; 24 h)		↓	TNF-α decreased butyrate uptake and beta-oxidation TNF-α decreased MCT1 and BCRP mRNA levels	
MCT1	Inflamed colonic mucosa of patients with active UC and CD (vs. non-inflamed mucosa and control subjects)		↓	Decrease in MCT1 mRNA and protein levels The decrease in MCT1 levels is > than in non-inflamed colonic mucosa	[35]
	Rats with DSS-induced colitis		↓	Decrease in MCT1 mRNA and protein levels	
	HT-29 cells/TNF-α (30–100 ng/mL), IFN-γ (300–1000 U/mL) (24 h)		↓	TNF-α and IFN-γ decrease butyrate uptake and oxidation TNF-α and IFN-γ decrease MCT1 mRNA and protein abundance TNF-α and IFN-γ decrease MCT1 gene transcription and had no effect on MCT1 mRNA stability	
SMCT1	Inflamed colonic mucosa of patients with active UC and CD (vs. non-inflamed mucosa)		=	No change in SMCT1 mRNA levels	[36]
SMCT1	IEC-6 cells and Caco-2 cells/TNF-α (5–10 ng/mL) (24 h)		↓	TNF-α decreases SMCT1-mediated butyrate transport and SMCT1 mRNA levels in IEC-6 cells TNF-α decreased SMCT1 promoter activity in Caco-2 cells	[37]
	Mice with DSS-induced colitis		↓	Decrease in SMCT1 mRNA and protein levels	
MCT1	Mice with DSS-induced colitis		↓	Decrease in MCT1 mRNA levels	[38]
MCT4			↑	Increase in MCT4 mRNA levels	
MCT1	Mice with T cell transfer-induced colitis		↓	Decrease in MCT1 mRNA levels	[39]
SMCT1			↓	Decrease in SMCT1 mRNA levels	
MCT1	Mice with DSS-induced colitis		↓	Decrease in MCT1 mRNA levels	[40]
MCT1	Caco-2 cells/TNF-α, IFN-γ (100 ng/mL) (24 h)		↓	Decrease in MCT1-mediated butyrate transport No change in MCT1 mRNA levels	[41]
MCT1	Caco-2 cells/TNF-α, INF-γ (200 ng/mL) (24 h)		↓/↑	TNF-α and INF-γ decrease MCT1-mediated transport of butyrate (10 μM) INF-γ increases MCT1-mediated transport of butyrate (10 mM)	[42]
MCT1	Ex vivo porcine colonic tissue/TNF-α (50 ng/mL; 1 h) Caco-2 cells/TNF-α (25–100 ng/mL; 1 h)		↓	Decrease in MCT1 mRNA levels	[43]
MCT1	Primary rat colonic epithelial cells/TNF-α (500 pg/mL) (24 h)		↓	Decrease in MCT1 mRNA levels	[44]
MCT4			↓	Decrease in MCT4 mRNA levels	
MCT1	HT-29 cells/TNF-α (50 ng/mL), IFN-γ (100 ng/mL) (24 h)		↓	Decrease in butyrate uptake and oxidation No effect on MCT1 mRNA levels	[45]
MCT1	NCM460 cells/indirect coculture with M1 human macrophages (24 h)		↑	Increase in MCT1 mRNA levels; this effect is Nrf2 activation-dependent	[20]
MCT4			↓	Decrease in MCT4 mRNA levels	
MCT1	Ex vivo calves’ ruminal epithelial tissue/LPS		=	No change in MCT1 mRNA levels	[46]
SMCT1				No change in SMCT1 mRNA levels	
BCRP	Inflamed colonic mucosa of patients with active CD and UC (vs. non-inflamed mucosa and control subjects)		↓	Decrease in BCRP protein levels The decrease in BCRP levels is > than in non-inflamed colonic mucosa No change in BCRP mRNA levels The effect is mediated by inflammation-dependent activation of the unfolded protein response	[47]
BCRP	Inflamed colonic mucosa of patients with active UC (vs. non-inflamed mucosa and control subjects)		↓	Decrease in BCRP mRNA levels The decrease in BCRP levels is > than in non-inflamed colonic mucosa	[48]
BCRP	Inflamed colonic mucosa of patients with active UC (vs. non-inflamed mucosa)		↓	Decrease in BCRP mRNA levels	[49]
MCT4	Colonic mucosa of patients with active CD and UC (vs. non-inflamed mucosa and control subjects)		↑	Increase in MCT4 protein levels The increase in MRP4 levels is > than in non-inflamed colonic mucosa	[50]
MCT4	Colonic mucosa of patients with active UC (vs. control subjects) Mice with DSS-induced colitis		↑	Increase in MCT4 protein levels	[51]

Abbreviations: BCRP: breast cancer resistance protein; CD: Crohn’s disease; DSS: dextran sulfate sodium; ERK1/2: extracellular signal-regulated kinases 1 and 2; IFN-γ: interferon-γ; LPS: lipopolyssacharide; MCT1: monocarboxylate transporter 1; MCT4: monocarboxylate transporter 4; Nrf2: nuclear factor erythroid 2-related factor 2; ROS: reactive oxygen species; SMCT1: sodium-coupled monocarboxylate transporter 1; tBOOH: *tert*-butylhydroperoxide; tBHQ: *tert*-butylhydroquinone; UC: ulcerative colitis; TNF-α: tumor necrosis factor-α.

## 4. Regulation of Butyrate Transport by Inflammation

**Figure 2:**
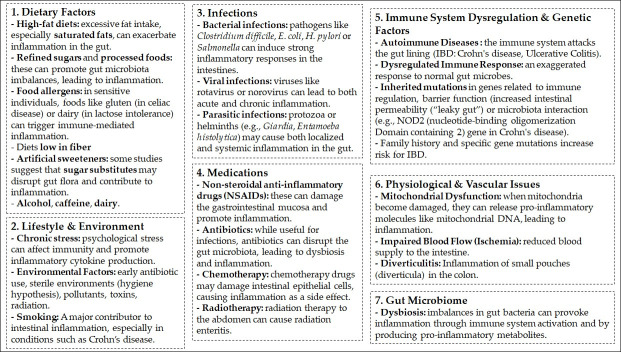
Major causes of intestinal inflammation.

Intestinal inflammation has a complex and multifactorial etiology, resulting from a breakdown in the delicate homeostatic balance between the host’s immune system, the gut microbiota, and various environmental factors. Sources of acute or chronic inflammation at the intestinal level include host genetic predispositions (e.g., mutations in genes that affect immune function and the mucosal barrier), disruptions in gut microbiota composition (dysbiosis), infectious agents (including bacterial, viral, and parasitic pathogens), ischemia, radiation enteritis and environmental factors such as Western diets high in processed fats and sugars, poorly tolerated fermentable carbohydrates, non-steroidal anti-inflammatory drugs (NSAIDs), certain food additives, antibiotic use, and smoking [52,53] (Figure 2).

Inflammatory bowel diseases (IBDs) are a group of life-threatening chronic diseases of the gastrointestinal tract characterized by episodes of intestinal inflammation, with Crohn’s disease (CD) and ulcerative colitis (UC) being the most significant. The incidence and prevalence of IBD, an immune-mediated condition, markedly increased over the second half of the 20^th^ century, and since the beginning of the 21^st^ century, IBD has been considered one of the most prevalent gastrointestinal diseases with accelerating incidence in newly industrialized countries [54,55]. The etiology and pathogenesis of IBD is multifactorial, with genetic susceptibility, environmental factors such as tobacco or NSAID use, gut microbiota dysbiosis, barrier dysfunction, immune dysregulation (with increased levels of proinflammatory cytokines such as interferon-gamma (IFN-γ) and tumor necrosis factor-alpha (TNF-α)), and increased oxidative stress levels being involved [56,57].

A summary of the effect of inflammation/inflammatory mediators on intestinal butyrate transporters is shown in Table 1.

The inflamed intestinal mucosa of patients with IBD is characterized by reduced butyrate metabolism (oxidation), associated with a decrease in butyrate cellular uptake through MCT1 downregulation [32,33,34,35]. Of note, MCT1 is downregulated in the inflamed mucosa to a greater degree than in non-inflamed mucosa of UC and CD patients [33,35]. Interestingly, infliximab (an anti-TNF-α monoclonal antibody) markedly increased *MCT1* mRNA levels in the inflamed colon of infliximab-responsive UC patients [32]. In relation to SMCT1, information is scarce, but no alteration in *SMCT1* mRNA levels was reported in the inflamed colon of UC patients [36].

Additionally, a decrease in *MCT1* and *SMCT1* gene expression is observed in colitis-induced rodent models [35,37,38,39,40].

The inhibitory effect of an inflammatory environment on butyrate uptake at the intestinal level appears to result from the action of the proinflammatory cytokines TNF-α and IFN-γ, which have been shown to reduce butyrate uptake and oxidation in intestinal epithelial cell lines in vitro and colonic epithelial organoid cultures and colonic tissue ex vitro [34,35,37,41,42]. However, these effects may depend on the luminal concentration of butyrate. Indeed, our group verified that, although uptake of BT (10 µM) by Caco-2 cells was inhibited by both TNF-α and INF-γ, INF-γ increased uptake of a high concentration of butyrate (10 mM) in these cells [42]. Besides TNF-α and IFN-γ, IL-1β also appears to reduce butyrate oxidation in an intestinal epithelial cell line [58].

Multiple studies strongly suggest that the inhibitory effect of these two proinflammatory cytokines on butyrate uptake results from downregulation of *MCT1* and *SMCT1* gene expression [34,35,37,43,44]. Interestingly, while TNF-α treatment decreased both *SMCT1* mRNA expression levels and butyrate uptake by intestinal epithelial cells (IEC-6 cells), pretreatment with *Lactobacilli plantarum* significantly attenuated the inhibitory effects of TNF-α on SMCT1 [37]. Reduced colonic expression of *SMCT1* and butyrate transport were also found in spontaneously hypertensive rats, a model presenting with high colonic TNF-α levels [59,60]. However, two reports did not find an effect of TNF-α and IFN-γ on *MCT1* mRNA expression levels [41,45].

So, while most available data show that the decrease in butyrate uptake by inflammatory cytokines is probably related to a reduction in the transcription rates of *MCT1* and *SMCT1*, more investigation is needed in order to confirm this. Specifically, investigating whether these cytokines influence the transporters posttranscriptionally—through modulation of membrane insertion or intracellular signaling-dependent regulation—would be of particular interest.

However, an opposite effect of inflammatory mediators on *MCT1*, namely an induction, was described by Diehl et al. [20]. In this work, it was verified that indirect coculture of a normal human intestinal epithelial cell line (NCM460) with M1 (classically activated), but not M2, human macrophages induced Nrf2 activation-dependent *MCT1* expression. In contrast, *MCT4* expression was reduced. It was also verified that Nrf2 knockdown or ROS scavenging blocked these coculture effects in NCM460 cells [20]. However, it must be pointed out that the effect of pure cytokines was not evaluated; instead, an indirect coculture experimental model was used, in which NCM460 cells were exposed to M1 macrophage secretions, which are known to include high levels of proinflammatory cytokines and chemokines such as TNF-α, interleukin-6 (IL-6), interleukin-1β (IL-1β), interleukin-12 (IL-12), and C-X-C motif chemokine ligand 10 (CXCL10), among other substances [61]. It is thus possible that other compounds secreted by M1 macrophages, besides TNF-α, are also able to affect *MCT1* gene expression, thus explaining the overall stimulatory effect observed.

In contrast, no significant effect on butyrate transport activity and *MCT1* and *SMCT1* gene expression occurred after ex vivo exposure of calves’ ruminal epithelial tissue to lipopolysaccharide (LPS), a structural component of the outer membrane of Gram-negative bacteria that acts as a potent trigger of the inflammatory response [46]. A contrasting effect of TNF-α and LPS on butyrate transporters was also verified in an ALS cell line model (mutant motor neuron-like NSC-34 cell line), where TNF-α increased *SMCT1* mRNA levels and transport activity, whereas LPS caused an opposite effect on both *SMCT1* mRNA levels and activity [23].

As mentioned before, BCRP and MCT4 also modulate butyrate intracellular concentrations, because BT is a substrate of these two transporters at the colonic epithelial level. However, information on the influence of inflammation on these two transporters is scarce.

In relation to BCRP, the mRNA and protein levels of this ABC transporter were found to be strongly reduced in the inflamed intestine and colon of IBD patients, not only in relation to control subjects but also in relation to biopsies from non-inflamed mucosa of IBD patients [33,34,47,48,49]. Moreover, the inflammatory mediator nitric oxide was described to activate the unfolded protein response and concomitantly reduce the plasma membrane localization as well as the transport function of ABCG2 [47].

As for MCT4, analysis of human data shows that the expression of MCT4 is upregulated in IBD [50,51], being the same trend observed in rodent models of colitis [38,51]. However, an opposite effect was found with primary rat colonic epithelial cells, where TNF-α was able to reduce *MCT4* mRNA levels [44].

Although regulation of butyrate transporter expression has been mostly studied in vitro in intestinal epithelial cells, LPS and TNF-α were reported to induce *MCT1* mRNA and protein expression in mouse peritoneal and J774.1 macrophages [62]. On the basis of this report, inflammation appears to have opposite effects on MCT1-mediated transport of butyrate in colonic intestinal epithelial cells and peritoneal inflammatory macrophages.

Finally, the effect of NSAIDs upon MCT1 and SMCT1 was previously investigated in a few studies. MCT1 activity is inhibited by several NSAIDs (including salicylic acid, acetylsalicylic acid, diflunisal, diclofenac, ketoprofen, indomethacin, and naproxen) [63,64,65,66,67,68,69]. In contrast, our group verified that butyrate uptake by Caco-2 cells (mainly MCT-1 mediated) was stimulated by acetylsalicylic acid [43]. As for SMCT1, this transporter is also inhibited by some NSAIDs (including ibuprofen, ketoprofen, fenoprofen, and naproxen) [70,71,72,73] while stimulated by others (diclofenac, meclofenamate, and sulindac) [74]. These distinct effects of several NSAIDs on both MCT1 and SMCT1 point to the possibility that their distinct effects are related to other effects than their anti-inflammatory effect.

## 5. Relevance of Redox Modulation of Butyrate Transport on Butyrate-Mediated Effects

The beneficial effects of butyrate at the intestinal level include anti-inflammatory and anticarcinogenic actions. Accordingly, a protective effect of this SCFA against CRC and IBD, two of the most significant intestinal diseases affecting humans, due to their high prevalence and substantial impact on morbidity and mortality, is well established [1,2,75].

CRC is a malignant disease that develops from the uncontrolled growth of abnormal cells in the lining of the colon or rectum. In 2023, CRC constituted the third most common cancer worldwide, accounting for approximately 10% of all cancer cases, being the second leading cause of cancer-related deaths worldwide [76]. As for IBD, as already stated, it is a chronic inflammatory disease of the gastrointestinal tract, requiring lifelong medication and often causing significant morbidity [54,55]. These two intestinal diseases are linked, as chronic inflammation is known to promote carcinogenesis, and patients with IBD are at increased risk for developing CRC [77,78]. This is clearly demonstrated by the fact that although IBD-related CRC is responsible for only approximately 2% of the annual mortality from CRC overall, CRC is responsible for 10–15% of annual deaths in IBD patients [79].

Importantly, butyrate transporters are altered in these two diseases. In IBD, a downregulation of MCT1 and SMCT1 is observed, highlighting the essential contribution of MCT1- and SMCT1-mediated butyrate transport into colonocytes for its anti-inflammatory activity (see Section 4). In relation to CRC, a downregulation of MCT1 (in the early stages of carcinogenesis) and SMCT1 is observed [2,5,6], supporting a crucial role of MCT1- and SMCT1-mediated butyrate uptake by colonocytes for its anticarcinogenic effects. Accordingly, colonic MCT1 and SMCT1 have been proposed to function as tumor suppressors [2,80,81]. However, although downregulation of MCT1 is observed in initial stages of CRC development, a later upregulation of MCT1 in advanced metastatic CRC tumors has been described [82,83,84], possibly providing a route for excess lactate produced by CRC cells to leave the cell [84]. As for BCRP, its mRNA and protein expression are significantly downregulated in human colorectal adenomas and CRC and in mouse models of CRC, suggesting that malignant transformation of the colonic epithelium in vivo is accompanied by a significant downregulation of BCRP. However, similarly to MCT1, some studies described BCRP overexpression in invasive CRC (i.e., advanced stage of carcinogenesis) [85,86,87].

As mentioned above, the molecular targets for butyrate at the intestinal level are located on the cell surface as well as inside cells. More specifically, both in colonic epithelial cells and mucosal immune cells, three cell-surface G-protein-coupled receptors, GPR41, GPR43, and GPR109A, are targets for butyrate [1,2]. Because these receptors are present in the lumen-facing apical membrane of colonic epithelium, luminal butyrate is able to stimulate these receptors without the need to enter the cells. In addition to these extracellular actions, butyrate also has intracellular actions (HDAC inhibition), which obviously requires its uptake into colonic epithelial cells. Furthermore, butyrate needs to traverse the colonic epithelial cells to the serosal side to act on the immune cells present in the lamina propria. Therefore, butyrate transporters expressed in colonic mucosa are major determinants of the anticarcinogenic and anti-inflammatory beneficial effects of this SCFA [1,2,6]. Some works clearly demonstrate this relationship. For instance, Thangaraju et al. verified that re-expression of SMCT1 in SW480 CRC cells, in which SMCT1 was completely silenced, restored butyrate transport and induced apoptosis, but only in the presence of butyrate [88]. Moreover, our group verified that the bile salt chenodeoxycholic acid, which was able to inhibit both MCT1- and SMCT1-mediated butyrate uptake by IEC-6 cells, reduced the cytotoxic effect and the prodifferentiation effect of this compound [19]. Finally, we also verified that inhibition of BCRP significantly potentiated the inhibitory effect of butyrate upon intestinal epithelial IEC-6 cell proliferation [7].

In this context, a few works investigated the consequences of changes in butyrate transporter function induced by redox modulators or inflammatory mediators on its anticarcinogenic and anti-inflammatory actions.

In relation to the effect of redox modulators on the colonic effects of butyrate, given the important protective roles played by butyrate at this level against CRC and IBD, it is plausible to conclude that a decrease in cellular butyrate uptake caused by increased oxidative stress levels, as observed by our group [18], will obviously contribute to the procarcinogenic and proinflammatory effect of oxidative stress at this level. However, this has not been yet investigated. Indeed, the effect of redox status has been investigated to date only in a few works that tested the effect of polyphenols on butyrate actions at the colonic level. Dietary polyphenols are a large family of compounds with antioxidant, anti-inflammatory, and anticarcinogenic characteristics [89]. An interesting work described that long-term (48–72 h) treatment of HT-29 CRC cells with the polyphenols epicatechin (EC) (100 µM) or epigallocatechin-3-gallate (EGCG) (20 µM) reduced butyrate-induced cellular differentiation, associated with a reduction in the cellular uptake of butyrate. Importantly, it was also verified that this effect was not related to changes in *MCT1* expression but rather to its membrane localization [90]. The effect of these two polyphenols in another colorectal cancer cell line (Caco-2 cells) was, however, rather distinct. EC did not show a consistent effect upon butyrate uptake and EGCG decreased butyrate uptake in the short term (23 min; 10 µM) but increased it in the long term (48 h; 1–10 µM). Surprisingly, the increase in butyrate uptake induced by EGCG was associated with a decrease in butyrate oxidation and *MCT1* mRNA levels. Moreover, EGCG did not interfere with the effect of butyrate on Caco-2 cell viability, cell proliferation, differentiation, and apoptosis [91]. The differences between the results of these two works might be related to the fact that distinct CRC cell lines were used, because similar exposure times and concentrations of the polyphenols were used. Moreover, it is also important to mention that no evidence was presented showing that the effect of these polyphenols on butyrate transport and butyrate-induced cellular effects is related to redox alterations only. Although the anticarcinogenic effect of polyphenols is strongly related to their redox modulating effect, these compounds possess many other biological activities (e.g., they are anti-inflammatory, epigenetic modulators and they interfere with cell signaling pathways and with mitochondrial function) that may contribute to their anticancer effect [92,93]. Moreover, numerous studies show beneficial effects of flavonoids as potent antioxidants under normal conditions but as having potent pro-oxidant properties in cancer cells [92]. So, direct evidence linking redox modulation by these compounds to changes in butyrate transporter function and subsequent biological outcomes is currently lacking and constitutes a knowledge gap.

In relation to inflammation, our group verified that, in Caco-2 cells, the antiproliferative effect of BT, but not its cytotoxic effect, is MCT1-dependent. In these same cells, we observed that the antiproliferative effects of butyrate and of proinflammatory cytokines (which inhibited MCT1-mediated butyrate uptake) were not additive, evidencing a common cellular molecular target. In contrast, the cytotoxic effects of BT and of the proinflammatory cytokines were additive [41]. This clearly links an inflammatory environment, and more specifically the proinflammatory cytokines TNF-α and IFN-γ, to a decrease in MCT1-mediated colonic uptake of butyrate and, consequently, to a decrease in its beneficial effects in relation to CRC.

Cyclooxygenase-2 (COX-2) overexpression plays an important role in the inflammation–carcinogenesis pathway of CRC [94] and it is well established that the regular use of NSAIDs exerts a protective effect against CRC development [95]. In this context, we verified that acetylsalicylic acid stimulated butyrate uptake and potentiated its antiproliferative effect, suggesting that the potentiation of the anticarcinogenic effect of BT in Caco-2 cells is related to the increase in its cellular uptake [42].

## 6. Conclusions and Future Perspectives

In conclusion, the existing evidence points to the conclusion that all the butyrate transporters at the intestinal mucosal level (MCT1, SMCT1, MCT4, and BCRP) can be modulated both by redox and inflammatory status. A role of Nrf2, TNF-α, and IFN-γ in mediating the effect of oxidative stress and inflammation, respectively, on MCT1 and SMCT1 is suggested. Moreover, colonic inflammation/TNF-α and IFN-γ appear to negatively impact MCT1, SMCT1, and BCRP, while positively impacting MCT4. This suggests that these conditions will be associated with decreased intracellular levels of butyrate, which will result in a diminution of the protective effects of this compound at the colonic level.

However, for all transporters, the evidence is very scarce and inconsistent. So, more investigation is needed that specifically can address the effect of distinct ROS species and antioxidant mechanisms and their mode of action in regulating the activity of these transporters. Similarly, additional work examining in greater depth the effect of distinct inflammatory modulators and their specific mode of action resulting in changes in butyrate activity is also needed. Specifically, it would be important to investigate the effects of oxidative stress and inflammatory cytokines at the posttranslational level, because the activity and membrane location of these transporters are regulated both by phosphorylation/dephosphorylation and by intracellular signaling pathways such as nuclear factor κ-light-chain-enhancer of activated B cells (NF-κB), mitogen-activated protein kinase (MAPK), and Janus kinase/signal transducer and activator of transcription (JAK/STAT), which are known molecular targets of both oxidative stress and inflammation [96]. Knowledge on regulation of butyrate transport mechanisms at the colonic level by oxidative stress and inflammation is very important, given the important beneficial role of this SCFA at that level and the fact that increased oxidative stress levels and inflammatory status are present in a series of intestinal conditions and pathologies. In this context, it would also be important to compare the effects of acute and chronic oxidative stress and inflammation, which are relevant from a medical point of view. Additionally, further work investigating the link between changes in butyrate transport and its beneficial effects at the colonic level would certainly help to clearly establish these transporters as potential cellular targets in CRC and IBD.

## Data Availability

No new data were created or analyzed in this study. Data sharing is not applicable to this article.

## Abbreviations

The following abbreviations are used in this manuscript:

ALS	Amyotrophic lateral sclerosis
BCRP	Breast cancer resistance protein
BT	Butyrate
CRC	Colorectal cancer
GPR	G-protein-coupled receptors
HDACs	Histone deacetylases
IBD	Inflammatory bowel disease
IFN-γ	Interferon-gamma
LPS	Lipopolysaccharide
MCT1	Monocarboxylate transporter 1
MCT4	Monocarboxylate transporter 4
NSAIDs	Non-steroidal anti-inflammatory drugs
NRF2	Nuclear factor erythroid 2-related factor 2
ROS	Reactive oxygen species
SCFA	Short-chain fatty acid
SMCT1	Sodium-coupled monocarboxylate transporter 1
TNF-α	Tumor necrosis factor-alpha
